# Forms, interactions, and responses to social support: A qualitative study of support and adherence to photoprotection amongst patients with Xeroderma Pigmentosum

**DOI:** 10.1111/bjhp.12396

**Published:** 2019-11-22

**Authors:** Jessica Walburn, Rebecca Anderson, Myfanwy Morgan

**Affiliations:** ^1^ Faculty of Life Sciences and Medicine School of Cancer and Pharmaceutical Sciences King’s College London UK

**Keywords:** adherence, disclosure, emotional support, photoprotection, practical support, qualitative, social support

## Abstract

**Objectives:**

Social support influences adherence to treatment in chronic illness, but there is uncertainty about its facilitators and constraints. This study explored the forms, processes, and responses associated with mobilization of informal support across three life contexts amongst patients with Xeroderma Pigmentosum (XP), a condition requiring rigorous photoprotection to reduce cancer risks.

**Design:**

Qualitative interview study.

**Methods:**

A total of 25 adults with XP participated in semi‐structured interviews conducted face to face. An inductive thematic analysis was applied using a framework approach.

**Results:**

Practical support, involving both assistance with recommended photoprotection and adjusting daily activities to reduce exposure, was the key form of support provided by family and friends. However, responses to this support differed with two groups identified based on the relative priority given to photoprotection in daily life and processes of disclosure. For ‘positive responders’, support aligned with their own priorities to photoprotect, conveyed feelings of being cared‐for and was facilitated by talking openly. In contrast, for ‘negative responders’ support conflicted with their priority of living ‘normally’ and their limited disclosure hindered receipt of helpful support in personal, clinic, and work interactions. Fears of workplace stigma also reduced disclosure amongst participants open in other contexts.

**Conclusions:**

Practical support conveyed psychosocial support with positive effects on adherence. This suggests the traditional separation into practical and emotional support is overly simplistic, with measures potentially missing important aspects. Interactional processes contribute to the effects of support, which can be addressed by targeting disclosure, stigma, and other barriers at individual and organizational levels.

**Statement of contribution**

***What is already known on this subject?***

- Social support can be both a facilitator and a hindrance to treatment adherence.
- Practical support is identified as the most important form of support in the context of adherence.
- The processes of support underpinning its relationship to adherence are unclear.

***What does this study add?***

- Variations in the provision and impacts of support are influenced by participants’ disclosure and attitudes to photoprotection, with two key groups comprising ‘positive responders’ and ‘negative responders’.
- The influence of emotional support on adherence may be underestimated through neglect of the ways in which practical support often conveys feelings of being valued and cared‐for.
- Barriers to mobilizing effective adherence support extends across life spheres, with fears of stigma and discrimination in work settings highlighting the need to intervene at individual and organizational levels.

## Background

Poor adherence to treatment is a continuing barrier to optimal health care outcomes, with estimates indicating that up to 50% of medications prescribed to people with long‐term conditions (LTCs) are not taken as prescribed (WHO, 2003). Rates of adherence for recommended behavioural changes (e.g., diet, physical activity, photoprotection) are even lower (Khan & Socha‐Dietrich, 2018). Adherence studies have reported moderately positive correlations between instrumental or practical support (behavioural or material assistance with tasks) and adherence, with weaker associations for emotional support [demonstrations of love, caring, sympathy (Thoits, 2011)] (DiMatteo, 2004; Scheurer, Choudhry, Swanton, Matlin, & Shrank, 2012). However, despite these associations we know little about ‘how’ support may influence adherence. Psychological models employed to understand adherence, such as the Common‐Sense Model of illness (Leventhal, Meyer, & Nerenz, 1980) and the Health Action Process Approach (Schwarzer, 2008), acknowledge the importance of social influence but the processes involved have not been examined. Responding to this gap, condition‐specific reviews of support and adherence (Ladin, Daniels, Osani, & Bannuru, 2018; Miller & DiMatteo, 2013) and the wider support literature recommend that qualitative research is required to focus on the mechanisms leading to the observed associations between support and adherence (Thoits, 1995, 2011; Vassilev *et al.*, 2011).

As part of a programme of mixed‐methods research to improve adherence to photoprotection amongst people with Xeroderma Pigmentosum (XP; see Walburn *et al.*, 2017), a very rare genetic condition, we identified gaps in the understanding of the influence of social processes on adherence. Patients with XP are required to constantly follow an extremely demanding photoprotection regime because they are unable to repair DNA damage to the skin caused by exposure to ultraviolet radiation (UVR) in daylight (Sethi, Lehmann, & Fassihi, 2013). This involves wearing a transparent face visor or other protective head/face covering, UVR protective clothing, glasses, and regular use of a broad‐spectrum sunscreen, together with reducing time spent outside and exposure to daylight (Fassihi, 2013). Although following this regime is the only way to avoid the potentially life‐threatening skin and eye cancers (Bradford *et al.*, 2011), adherence can be poor (Walburn *et al.*, 2019).

As little is known about social influences on photoprotection, there is a need for a rich contextual understanding of how social support might ‘work’ in people’s daily lives to inform an effective intervention. We selected a qualitative approach, which is ideally suited to studying under‐researched topics, and can provide explanations as to ‘how’ a phenomenon operates (Green & Thorogood, 2004). This study of adults with XP explored the role of support in adherence to photoprotection by investigating support interactions from the perspective of the recipient. It focused on the forms of support provided, the influence of patient–supporter interactions and responses to support. The study also examined supportive relationships provided not only by family and friends, but also encountered in the spheres of work and the XP clinic.

## Methods

### Design

A qualitative study with individual semi‐structured interviews conducted with adults diagnosed with XP. The study was approved by Camden & Kings Cross Research Ethics Committee 15/LO/1395, London, UK.

### Recruitment process

Patients were eligible to participate if they were diagnosed with XP and received their care from the UK’s only XP specialist multidisciplinary service at Guy’s and St Thomas’ NHS Foundation Trust. Patients included were aged 16 years and over, were not cognitively impaired, spoke English adequately to take part in an interview, and had not opted out of taking part in any research. Eligible adults (*n* = 38) were identified by a consultant dermatologist from the clinic register and sent an information sheet with a covering letter detailing the aims of the study and how to contact the study team. After two weeks, patients were phoned by a research nurse to ask whether they would like to take part or, if due to attend the clinic, they were approached during their routine appointment. Twenty‐five patients decided to participate. Of those who did not take part, seven could not be contacted and six declined to participate (4 men and 2 women), explaining that they did not have enough time or did not want to think about XP.

### Characteristics of participants

We interviewed 17 male and 8 female participants aged between 16 and 63 years from a variety of ethnic groups. Of these, 13 had Asian backgrounds, which reflected the higher prevalence of XP in communities from the Middle East (Fassihi, 2013). The gender balance was indicative of the higher proportion of adult male patients registered with the XP clinic (26 men and 17 women). Thirteen were married or living with a partner. The sample was diagnosed with a range of XP genetic complementation types, and 11 participants had experienced skin cancer. To protect anonymity, we do not report the characteristics of each participant and new study numbers were allocated.

### Procedure and materials

All interviews were carried out between February and June 2016. All were conducted in patients’ homes, except for a single telephone interview due to the participant being abroad. Two non‐medical researchers (RA/JW), experienced in qualitative interviewing, separately conducted the interviews and were accompanied on the home visit by a research nurse. The researchers and nurse were not known to the participants. Written informed consent was obtained by the nurse, who was not present for the interview. The interviews lasted for about an hour and were steered by a topic guide designed to elicit the range of experiences associated with living with XP, managing the required photoprotection and their experiences of social support (see Table 1). Interviews opened with broad ‘leading‐in’ questions designed to start to focus attention towards the potential challenges of living with XP, followed by narrower more specific topics with prompts as required. The topic guide provided a flexible framework to structure the discussion, but the direction of the interview was led by the participant.

**Table 1:** Excerpt from the topic guide for participants with XP

We know from talking to other people with XP that friends and family can help with photoprotection – do they help you or not?
How do they help? What do they do? Is there anything that they do that you don’t find helpful?
What sort of help do you get? Is it directly related to XP or more general support?
Is it enough to know that support is there if you need it?
Are there times when friends or family don’t help and make photoprotection more difficult?
How helpful have you found going to the XP clinic? In what way? (*explore clinical management of XP/role of clinic/other forms of support*)
Do different people give different sorts of support? (*explore sources of support, family, friends, work colleagues*)

### Analysis

Interviews were recorded with permission and transcribed verbatim by an independent transcribing agency. Anonymity was maintained by referring to the participant ID at the start of the recording. Transcripts were checked for accuracy with recordings by RA and then initially read and re‐read by all team members (RA/MM/JW). Analysis was inductive and based on a framework approach (Ritchie & Lewis, 2006). Transcripts were coded, and data were entered into NVivo 11 software. The main themes were then identified and summarized in a matrix, with cases as rows and themes as columns. This approach facilitates a constant comparative method as participants’ responses to a single theme can be viewed in the context of multiple themes, leading to the development of typologies and explanatory accounts (Gale, Heath, Cameron, Rashid & Redwood, 2013). Initially, team discussions were held to develop and refine emerging themes, which were then applied to the whole dataset by RA and reapplied by MM/JW as a check. Saturation of themes was achieved after eighteen cases. Next, detailed further refinement of themes took place (e.g., forms of support for photoprotection provided by family and friends) and subthemes identified (e.g., assistance for photoprotection practices before or during outdoor time). This further process of constant comparison culminated in the identification of a pattern of responses to social support common to two groups of participants, those who responded ‘positively’ or ‘negatively’. Further analysis of positive and negative responders was then undertaken to compare their experiences of support, interactions across contexts, and photoprotection activities. The analysis moved beyond description to generate explanatory interpretive accounts of the processes underpinning these responses. Mapping was used to facilitate the testing of hypotheses (e.g., How is disclosure linked to response to support provided by colleagues?) and to explore relationships between phenomena. Analysis was dynamic, moving back and forth from description to mapping.

The credibility of the analysis was supported by regular group review. For example, JW first categorized the participants as positive or negative responders and then justified groupings in response to questions and analysis by RA and MM. Any uncertainty about group membership initiated further discussion, a return to the data and continued review until an agreement was reached. A reflective diary was also kept during the analysis to keep concepts grounded in the data.

The credibility of the categorization of responses that emerged from the initial stages of analysis was assessed through a group discussion held with the two Clinical Nurse Specialists (CNS) from the XP clinical team. The CNS volunteered to take part. They were female, aged between 35 and 55, and have both worked with XP patients for over 5 years. We aimed to ascertain whether they recognized positive and negative responders from their clinical experience. This discussion provided additional insight into how, from the perspective of the HCP, the nature of responses to social support influenced support interactions at the clinic. We then returned to the data to consider whether there were differences in how negative and positive responders viewed the clinical interaction, which further informed our explanatory accounts. We gained written permission from the CNS to record the discussion and include quotations in the findings.

The findings and interpretation were also discussed with the Patient and Public Involvement (PPI) panel. Four PPI representatives (one patient, two parents, one teacher) participated in a discussion session. JW presented the forms of support with the different responses, and the group considered how the findings corresponded to their own experiences.

The Standards for the Reporting of Qualitative Research Guidelines (SRQR; O’Brien, Harris, Beckman, Reed, & Cook, 2014) were adhered to (see Table S1).

## Results

**Figure 1:**
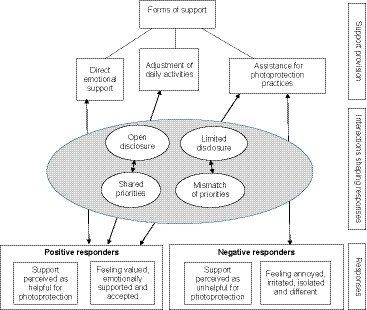
Themes, sub‐themes, and explanatory accounts underpinning responses to support provided by family and friends. [Colour figure can be viewed at http://www.wileyonlinelibrary.com]

The initial focus is on the experiences of the forms of support provided by family and friends, which are inherently positive. This is followed by an in‐depth analysis and explanatory account of processes underpinning responses to support, which included both positive and negative reactions (see Figure 1). We then examine social support responses within the wider social contexts of the XP clinic and work environments.

### Forms of support for photoprotection provided by family and friends

#### Assistance for photoprotection practices before or during outdoor time

Most participants spoke of photoprotection‐specific support being routinely offered by both friends and family. Practical support involved assisting directly with photoprotection tasks, such as giving reminders about applying sunscreen and wearing protective clothing.

They’re always reminding me, have you got your sunblock on, have you got your glasses? (no.1, female, 45 years).

Friends and family also monitored UVR risk ‘in the moment’, alerting patients to perceived danger and moving into shade if there was none:

If I’ve been in the shade and then suddenly, I walk through a sunny patch. Everyone will say quick, over here, and we’ll swap places (no.16, female, 63 years).

They’re all quite fussy…[about shade] and they’ll dig umbrellas out for me (no.23, male, 55 years).

Others described family members negotiating with strangers to enhance protection in public situations.

someone was leaning on the blind and it was staying up, and she just grabbed it and dragged it down……She said my brother needs this down (no.22, male, 26 years).

#### Adjustment of daily activities

Close family often reduced UVR risk through altering daily activities, including shifting activities to the evening and engaging in new roles such as taking on responsibility for outdoor chores or errands. As one man explained:

if we are going shopping during the day, then I’m the one who would normally be staying in the car……and the missus would actually go into the shops (no.10, male, 39 years).

People of all age groups described how friends would adapt daily activities to take account of their need to avoid UVR, including shifting social activities to different times of day or allowing extra time to apply protection. These adjustments facilitated better photoprotection and respondents appreciated that this was generally done subtly, with the minimum of fuss.

they just brush it off, like yeah that’s fine. As long as you’re safe, we’re happy to chill out and let you put it on (no.19, male, 18 years).

#### Feeling valued and emotionally supported

Feelings of support were described as bolstering individuals’ emotional state and confidence to photoprotect, helping to reduce the negative impacts of living with such a demanding regime. Participants rarely spoke directly about emotional support, although several explained that they felt ‘cared‐for’ by the adjustment of activities and photoprotection‐specific support. A teenager described how he felt about the reminders from his family:

It is helpful, and it just shows that they care, that’s it. (no.11, male, 16 years).

For younger participants, adjustments conveyed acceptance and contributed to participants feeling valued by their social circle:

Things they are organising, like dinners for their birthdays. It’s like right, we’ll go out at 9pm and then you can. It’s not like oh, then you can come. It’s like we’ll go at this time. They don’t need to say why or… I think that’s just good friends (no.22, male, 26 years).

When ‘direct’ emotional support was needed, it was sought from a select few with whom they felt most comfortable. Some participants explained that knowing help was available was enough to feel emotionally supported:

I have to say that’s a very important type of support for me to know that, that their help… I probably don’t use it much, but it’s really good to know that there’s someone I can talk to if I need to. (no.9, male, 38 years).

### Interactions: Processes and responses

#### Positive responders

Eighteen people across the age span (16–63 years) were categorized as ‘positive responders’. These participants spoke positively about the support from family and friends. They appreciated the practical assistance with its positive emotional ‘side‐effect’ of ‘feeling cared‐for’. This support enabled participants to better adhere to photoprotection and reduce daily UVR exposure.

Yeah, it’s good to have someone reminding you. It’s good to have the reminder there. Because you’re reminded, I don’t go outside unprotected and be at risk (no.17, female, 62 years).

Similarly, another woman talked about the support provided by her daughter*:*

if my daughter is around, if I’m around her, they do. Like now, go put your sunscreen on and whatever……… Yeah, it is helpful (no.25, female, 55 years).

Practical assistance was gratefully received, since it aligned with the recipient’s emphasis on the importance of photoprotection. Positive responders were aware of the risks of UVR and were convinced that photoprotection was necessary. As a younger man described:

Even on cloudy days it’s still harmful for us… I’m always clothed up, hooded up, sun cream, I’ve got over like 100 SPF on my face and then got my cap (no.7, male, 27 years).

Positive responders’ support from friends was facilitated by their own willingness to freely disclose their condition and photoprotection needs.

I tell everything….like how every year I go to London to see the whole specialist team….tell them about my window being protected. I tell them how my life has been affected because of XP (no.11, male, 16 years).

Those who had been living with the condition for many years explained that friends and family knew all about their photoprotection needs:

So I think most people know about it. Yeah, when I’m talking about it or in conversation I say oh, I’ve got to put on my, find my glasses. I’ve got to do this, put, you know, most of my friends know that’s my regime, or what’s going on…… (no.16, female, 63 years).

Newly diagnosed positive responders wanted others to understand the rationale behind their photoprotection activities: As one participant explained:

…generally if I go out with my friends, and I’ve said to them I want to be in the shade. ……I’ve always said to them that it’s just because of my skin condition and I need to be in the shade, whereas my skin doesn’t, can’t repair myself as quick as, well, as you guys can… it’s DNA where my skin tends to basically not repair certain things, whereas …you can go out in the sun and not worry… (no.4, male, 36 years)

The degree of openness varied. Some initiated conversation about XP, whereas most responded to questions about their photoprotection behaviour.

I look very different with the visor on so it’s not surprising that people want to know why. So if someone wants to ask a question …I don’t mind answering (no. 13, male, 28 years).

#### Negative responders

Six younger participants were categorized as ‘negative responders’. This group comprised four men and two women, aged 20–37 years, of which four experienced a severe burning response to UVR. In contrast to the positive responders, they were reluctant to talk about their condition, did not find practical support helpful, and did not feel emotionally supported.

For these participants, reminders from both friends and family were annoying and inappropriate. They drew unwanted attention to a feeling of difference that participants wished to conceal. These reminders conflicted with their approach to living with XP, which was characterized by prioritizing being ‘normal’ over and above photoprotection.

I just think it’s a constant reminder that I’m different. So that’s why I don’t really like them [reminders to wear jacket when going out] (no.12, male, 21 years).

They were also internally conflicted about the need to protect; although aware of risks of UVR, they were reluctant to undertake photoprotection. As a younger participant explained:

There are times. Like usually it happens, I just think whatever. I just go out (without protection)… I know it’s bad,… (no.12, male, 21 years old).

In contrast to positive responders, they experienced strong feelings of difference associated with the visible damage to the skin from UVR and ‘standing‐out’ when photoprotecting:

My arms are full of freckles and my chest as well… I just want to be normal…You know, blemishes (freckles). Why am I so different? I look odd. I just look odd (no. 2, female, 35 years)

It’s like weird, I’m the weird person because I’d suddenly disappear because I can’t walk across the road (expose self to UVR) to buy stuff they’re buying. (no.15, male, 34 years).

Not talking much about XP to friends was a further defining characteristic of the negative responders. They would not explain their condition to friends as this respondent explained:

I’m not really good at talking, I just tell them it is a skin condition…I didn’t ask for it, it just happened. They don’t need to know (no.6, male, 20 years).

This group feared that being open would result in others thinking more negatively of them and would mean they had to deal with more questions and inappropriate comments. By limiting disclosure, these participants felt they were able to preserve their ‘normality’.

In a way it [not telling others] actually helps because then I’m just seen as normal rather than, oh you have to stay away kind of thing (no.12, male, 21 years).

Some explained that being secretive about XP had been encouraged by family members.

My dad was, no, don’t tell anybody about it. None of his family… Nobody knew (no.15, male, 34 years).

This group also spoke of instances where family and friends, who were not fully aware of their condition, actively encouraged them to take greater risks with UVR. Individuals were also sometimes mocked when they did use protection (e.g., application of sunscreen as effeminate). One patient described friends encouraging him not to wear protective clothing that would attract negative comments:

I put my hoody on but it looks strange and people say…Take that off, you’re making us look very dodgy and blah‐di‐blah (no.3, male, 37 years).

Others discouraged the use of the UVR metre, which is recommended to check UVR levels in new environments,

like if I was in the car with my sister, she would be like put it (UV meter) away, you’re going to keep going on about it. We’ve come out to relax and to be free (no.18, female, 31 years).

Some, particularly those who had not experienced skin cancer, felt that other friends or acquaintances did not view XP as a serious medical condition. They did not feel supported by their friends and men described a lack of empathy from their peer group.

I think it’s people’s lack of understanding. It’s such a non‐obvious, it’s such a subtle problem, right? If I have no leg, okay, people would see. Oh, yeah, just wear sun block, blah‐blah‐blah (no.15, male, 34 years).

Mainly it’s the men, they say oh you’re a man…deal with it you’ll be fine (no.3, male, 37 years).

Descriptions of feeling emotionally supported were notable by their absence. Negative responders described feeling isolated. As a younger man explained in response the following question:

Do you have any close friends that you feel understand XP? (Interviewer)

Not really. I’m an outsider. I don’t have that many friends anymore (no.6, male, 20 years).

The forms of support provided by family and friends, and explanatory processes underpinning responses are summarized in Figure 1. This illustrates the different levels of the support process and highlights the importance of the interactions between the recipient and the provider in shaping responses. Open disclosure and shared priorities were instrumental in facilitating a positive response to support that is offered, whereas concealment and mismatch of priorities left patients feeling isolated and unsupported. Hence, in contrast to negative responders, positive responders benefited from a broader range of support.

### Wider supportive relationships

#### Clinical staff at the XP service

Nearly all participants commented very favourably about the specialist XP clinic in terms of the information provided about XP and types of protection, given during their routine clinical appointment. However, there were differences in how positive and negative responders interacted with the staff, which influenced the quality of their relationship and the support they received. Positive responders described a proactive and collaborative relationship between themselves and the medical team, which extended the perception of support beyond what was received during the routine appointment (annual/biannual). They felt supported between appointments because they could contact staff if needed. As a participant who had been diagnosed nine years ago explained:

So if I’ve got problems with my skin condition, I just, I can email my nurse and she’ll help. (no.19, male, 18 years).

They actively sought assistance, freely disclosed, and welcomed staff following up their photoprotection resolutions made during appointments. As one participant explained in relation to the specialist nurse:

Makes me do things that I wouldn’t do. Call me on the phone and have you done that, have you done this? I never thought about window film, she said it’s important you do that. (no.21, male, 62 years)

As the nurses were aware of the patient’s life circumstances, they would go to great lengths to fit clinical care around the individual. Staff would facilitate photoprotection at work by helping to ensure that the environment was UVR safe and educating patient’s colleagues.

You wouldn’t phone that patient on that day, as that’s when they work (CNS B).

we assessed a whole university and worked out (using a UVR meter) where there was a safe area in each lecture room (CNS A).

Furthermore, if they knew of deficits in a patient’s informal support system, they would try to plug the gap.

I know that some patients don’t have the support network around them, so I’ll drop them an email or text to say good luck for your surgery, how did it go?. (CNS A)

In common with the practical support provided by family and friends, these forms of support contributed to patients’ feeling that they were cared‐for*.*

In contrast, the negative responders were not active partners in the management of XP and were not able to take advantage of the enhanced support available from the clinical team because they did not proactively disclose their needs. As one of the specialist nurses explained, certain patients did not actively engage; they limited contact and opportunity for support to the single appointment:

Some people just come to clinic and that is the only time they have XP. Even if you reach out to them during the year, you get nothing back. (CNS A)

Prioritization of ‘normality’ above XP requirements was a barrier to making best use of that routine clinic appointment, as a younger man admitted:

You just think I don’t want to be here, I want to get on with my life. Even though the doctors and everything are so nice, you don’t want to see them. (no.12, male, 21 years)

#### Colleagues at work

There were some examples of employers supporting patients to photoprotect at work. For example, one participant who was a teacher explained:

I also have to say the school, they timetable my classes in rooms that don’t get any sunlight so that’s helpful (no.9, male, 38 years).

However, several positive and negative responders explained that they actively concealed XP from those they worked with; they feared a negative reaction that might impact on opportunities at work and alter colleagues’ perceptions about what they were able to do. Fears of disclosure and subsequent lack of support were felt most keenly by negative responders, but some positive responders also described concerns about what others might think:

None of my colleagues know of my condition. My management do, but not people I see every day. I’m there putting my cream on and they [would be] giving me the funny looks. (no.10, male, 39 years).

He admitted that sometimes this stops him protecting.

People who were open with family and friends were also often more guarded at work. For example, an older office worker made light of his photoprotection needs:

you don’t want to be separate from the team, as such. You want to just be part of the normal everyday working life, as it were…. when they ask I say I find the sun a bit too strong and they just take it. (no.8, male, 63 years).

Some participants also feared an emotional over‐reaction, which would require further unwanted elaboration.

I had to go to make an appointment with my manager to say right, I’ve got to go through this treatment. And she started crying. Yeah, and she’s like I don’t want you to die. (no.24, female, 28 years).

Disclosure occurred only if essential, for example, requiring time off work for treatment. Little support was available for daily photoprotection needs and individuals generally managed UVR risk by altering their own behaviour.

So as soon as I come in they’ve got the blinds all up and I will put the blinds down when I get there. (no. 18, female, 31 years)

I always make sure my back is to the windows type of thing (no.2, female, 35 years).

### Credibility checks

The forms of support provided by family and friends were recognized by the parent and patient members of the PPI panel. No new aspects of support activities arose during discussions. Team discussions about initial groupings supported the existence of different support responses. Classification of participants as positive or negative responders was clear‐cut because the negative responders consistently described poor support experiences across different contexts. As expected, there was some within‐category variation, related specifically to the limiting of disclosure at work for some positive responders. The categorization of participants was endorsed by the clinical team.

## Discussion

This study draws attention to the variety of ways support is provided to participants with XP, alongside implications for photoprotection. Such support conveyed feelings of being cared‐for, which was important for many patients in coping with photoprotection behaviours that marked them out as different (Anderson, Walburn, & Morgan, 2017). The experience of emotional benefits from ostensibly practical photoprotection support highlights shortcomings of the ubiquitous separation of social support into distinct practical and emotional categories. Our findings indicate that it should not be assumed that practical support solely benefits adherence in terms of the enactment of the health behaviour (i.e., photoprotection). There is no consensus about what constitutes emotional support and the subtleties of how it is conveyed have rarely been researched (Kowitt *et al.*, 2015). It is hypothesized that conclusions about the stronger relationship between practical support and adherence, reported by quantitative studies (DiMatteo, 2004), have underestimated the role of emotional support. By using measures which focus on the *provision* of overt emotional support [e.g., The Social Support Inventory (Timmerman, Emanuels‐Zuurveen, & Emmelkamp, 2000); Significant Others Scale (Power, Champion, & Aris, 1988], they have missed the nuances of the *experience* of emotional support.

The study also highlighted (in terms of disclosure of XP and responses to the support offered) the importance of interactions between the support giver and receiver. These processes had important implications for photoprotection. Five out of six participants identified as negative responders in this analysis corresponded to those identified as strongly resistant to the photoprotection regimen with the lowest level of adherence in a prior qualitative analysis (see Morgan *et al*., 2019 for details of responses to photoprotection). The response to support depended on the extent to which both parties had shared values and priorities. Where support conflicted with the patient’s model of XP (e.g., where the patient prioritized normality and the provider emphasized UVR risk), support was rejected. Overt encouragement not to protect could also be linked to conflict between the patient’s medical model of XP and the ‘minor condition model’ of the peer group. Differences in perceptions of LTCs between patients and close relatives, or significant others, have been found to have deleterious effects in other conditions which require multiple self‐care behaviours (Pereira, Pedras, Machado, & Ferreira, 2016; Searle, Norman, Thompson, & Vedhara, 2007). Our findings reiterate early work by Revenson, Schiaffino, Majerovitz, and Gibofsky (1991) highlighting the existence of ‘problematic social support’ and contribute to understanding why a negative social environment is especially toxic (Helgeson & Zajdel, 2017). In this context, unhelpful practical support does not improve photoprotection; as a negative social interaction, it is also associated with greater distress (Wijnberg‐Williams, Kamps, Klip, & Hoekstra‐Weebers, 2006). This has been shown to independently hinder adherence (Kardas, Lewek, & Matyjaszczyk, 2013). We therefore speculate that negative social processes, by limiting social resources and amplifying the influence of other barriers, directly and indirectly impact adherence.

We have identified that disclosure is a prerequisite for accessing support, as depicted in models such as The Disclosure Process Model (Chaudoir & Fisher, 2010), and is also an ongoing mechanism by which support is tailored to be helpful. Without disclosure at work, there was little opportunity for helpful support. Even if personal and clinic support were still to be provided, without further disclosure or discussion, this would be unlikely to match the needs of the patient (cf. Cutrona & Russell, 1990). We speculate that an outcome of the disclosure process is an increased personalization of support. In terms of clinical care – by requesting support for issues that are most relevant, the engaged individual is benefiting from a more personalized condition management. This, in turn, is expected to be associated with better outcomes in adherence interventions (Allemann *et al.*, 2017).

Informed by the literature that disclosure itself reduces stigma by emotional processing (Beals, Peplau, & Gable, 2009), it is speculated that disclosure may improve adherence to photoprotection in two ways; it facilitates the receipt of helpful support (as above) and also diminishes feelings of shame (Clarke, Thompson, Jenkinson, Rumsey, & Newell, 2013), which is an additional internal barrier to adherence (Morgan *et al.*, 2019). However, there is an important caveat. Disclosure is only beneficial when the response is positive (Chaudoir & Fisher, 2010). In the work context, being open may have real personal costs in terms of loss of status, job roles, and even employment (Beatty, 2012). Fears of such outcomes are widespread in stigmatized conditions such as HIV (Cama, Brener, Slavin, & de Wit, 2017) and mental illness (Bos, Kanner, Muris, Janssen, & Mayer, 2009). This validates the work of the clinical team to educate and communicate accurate information about XP to employers in occupational settings. Reviews of the work environment emphasize the role organizations have in developing a ‘disclosure or stigma‐friendly culture’; it is not only behaviour change at the individual level that is required (Szeto & Dobson, 2010).

### Strengths and limitations

This study fills an important gap in the conceptual understanding of how social support influences adherence. This is especially important in the context of a disease where research has previously focused on biological, rather than psychosocial, influences on outcomes. We provide an in‐depth exploration which moves beyond description to explain phenomena. The trustworthiness of the reported positive and negative responses to support is significantly enhanced by the credibility checks, involving the XP clinical team and the PPI panel. However, the research context of the study and the rarity of XP constrained our approach. Given the demands of the wider programme of mixed‐methods research (Walburn *et al.*, 2017), we were limited to a single interview. We therefore focused on participants’ current experiences and did not examine how responses to social support may have changed over time with altered life circumstances and developments in the patient’s clinical condition. Future qualitative studies should explore responses to support amongst newly diagnosed XP patients. They should also examine patterns of responses to support during stressful periods, when explicit emotional support may be more important. Given that XP is a very rare condition and participants were taking part in a demanding mixed‐methods programme of research, due to fears of participant burden, it was also not feasible to undertake respondent validation.

### Implications

We identified a sub‐group of patients who do not experience helpful support from their family and friends, do not access it at work, and do not benefit fully from help available from their health care professionals (HCPs). We consider that these findings can inform intervention at the individual level to improve adherence to photoprotection in XP in the United Kingdom. To mobilize support, self‐management programmes need to raise the individual’s awareness of ‘support opportunities’, by encouraging self‐assessment of current support gaps and identifying ways in which others could assist with daily photoprotection requirements. For support to be a facilitator of adherence, barriers to perceiving it as helpful (i.e., enabling a positive response) should also be targeted. Negative responders would benefit from therapeutic strategies, such as linking life values with desired behaviour (i.e., better photoprotection; Zhang *et al.*, 2018), to reduce feelings of difference and promote acceptance. Exploration of the pros and cons of an individual’s current use of their network could be discussed, in conjunction with their willingness to disclose. Costs to the individual need to be fully considered, as a positive response to disclosure cannot always be assumed. Development of communication skills (e.g., active listening) would be beneficial. Whilst these recommendations may be relevant for global XP populations, further research is advised in non‐Western XP populations with different cultural contexts and health systems. Notwithstanding that experiences of support in XP are likely to have parallels with other photosensitive conditions, the extreme level of protection required suggests that the nature and pattern of responses could be different when less rigorous photoprotection is prescribed.

Beyond the individual, changes in the clinical and work environments are required. Clinicians already encourage significant others (family/friends) to be present at consultations. Based on findings that illness perceptions are modifiable (Jones, Smith, & Llewellyn, 2015), we recommend that HCPs also explore discrepancies and misunderstandings about XP and photoprotection. Incorporation of stigma‐reducing programmes at the occupational and societal levels, for example, campaigns promoting acceptance of staff with mental and physical health conditions (‘This is me’‐Barclays, n.d.) and visible difference initiatives (Changing Faces, n.d.), help to minimize environmental barriers that play a part in the poor mobilization of helpful support by the individual.

### Conclusions

By identifying modifiable processes and interactions that explain why social support can be both an enabler and obstacle to photoprotection, we confirmed the importance of moving from a narrowly patient‐focused approach to adherence in LTCs, to a more socially contextualized approach.

## Conflicts of interest

All authors declare no conflict of interest.

## Author contribution

JW was responsible for analysis and writing the paper. MM was responsible for study design and directed the research. RA and JW conducted interviews, checked transcripts, and were involved in the development of project materials and interpretation of findings. All authors approved the final paper.

## Supporting information


**Table S1.** The Standards for the Reporting of Qualitative Research Guidelines (SRQR) Checklist http://www.equator-network.org/reporting-guidelines/srqr/

## Data Availability

The data that support the findings of this study are not available due to privacy and ethical restrictions related to anonymity concerns associated with researching such a rare condition.
